# Temporal trends of alcohol and drug use among Inuit of Northern Quebec, Canada

**DOI:** 10.3402/ijch.v74.29146

**Published:** 2015-12-16

**Authors:** Marilyn Fortin, Richard E. Bélanger, Olivier Boucher, Gina Muckle

**Affiliations:** 1 School of Psychology, Laval University, Québec, Canada; 2 Population Health and Optimal Health Practices Research Unit, CHU de Québec Research Centre, Québec, Canada; 3 Department of Pediatrics, Centre mère-enfant Soleil du CHU de Québec, Laval University, Québec, Canada

**Keywords:** alcohol, marijuana, drugs, Inuit, indigenous communities

## Abstract

**Background:**

Alcohol and drug use is a serious health problem for many indigenous populations across Canada, including Inuit. The literature on substance use in these populations is too sparse to devise public health interventions.

**Objective:**

The present article portrays alcohol and drug use among Inuit living in Nunavik (Northern Quebec) between the 1990s and 2000s, and identifies socio-demographic characteristics related to substance use.

**Design:**

The Santé Québec Health Survey (1992) and the Nunavik Inuit Health Survey *Qanuippitaa* (2004) served as databases for this empirical work. Statistical comparisons were made of substance use variables in the 2 samples. Proportions were compared by chi-square tests (p≤0.05) with benchmarking of statistics for all of Quebec and, when available, all of Canada.

**Results:**

Alcohol and drug use among Inuit increased significantly between 1992 and 2004, particularly among young adults. Alcohol users consumed significantly more alcohol per drinking episode than other Canadians in both time periods. Considerable cannabis use was widespread. In 2004, no significant differences in frequencies of heavy drinking episodes were observed by gender, with 60% of drug users consuming alcohol on a regular basis.

**Conclusions:**

As in other populations from North America, this study profiles the increase in substance use among Inuit from Nunavik in the first part of the last 20 years. We observed distinct substance use patterns among them in comparison to other Canadians. Such findings, if replicated in the coming years, emphasize the need for major, culturally-relevant public health interventions in this population.

Alcohol consumption is widespread in Western societies. In Canada, in 2011, 82% of men and 74% of women reported that they drank alcohol at least once a month (1). Although the majority of Canadians usually consume less than 5 drinks per occasion, the drinking habits of some may elevate the risk of alcohol-induced problems (2–4). For instance, among Canadian drinkers in 2010, 1 in 5 men had more than 15 drinks per week or 3 drinks per day, and 1 in 8 had more than 4 drinks per occasion (1). Among women, 15% had more than 10 drinks per week or 2 drinks per day, and 1 in 10 had more than 3 drinks per occasion (1). Heavy drinking – for example, 5 drinks or more per occasion for men and 4 drinks per occasion for women at least once a month – is attributable to 5% of the worldwide global burden of disease (5) and 12.8% of healthcare costs in high-income countries (6), resulting from a number of associated short- and long-term health issues: foetal alcohol spectrum disorder among pregnant women, family problems, violence, accidents with injuries, heart disease, stroke, cancer, psychological distress and substance dependence (7–9). To limit the adverse effects of heavy drinking on long-term health, Canada's guide on alcohol and responsible drinking (10) recommends that women consume no more than 2 drinks per day and no more than 10 drinks per week, and men not more than 3 drinks a day and 5 drinks per week. The guide also recommends a limit of 3 drinks per day for women and 4 drinks a day for men to reduce negative effects in the short-term, and refrain from consuming on certain days in the week by both men and women to avoid alcohol tolerance.

For its part, cannabis is the illicit substance most widely consumed by Canadians (2, 4). As with alcohol, drug use is coupled with several social and health problems that vary according to the nature of the substance used, the amount consumed per episode of use and through which means (11, 12). For example, cannabis is tightly linked with psychosis (13, 14), whereas injection drug users are exposed to viral infections, such as hepatitis and human immunodeficiency virus (11, 15). Most substances used have the potential to reduce physical coordination, distort sensory perception and impair memory, attention and judgment. There is no question that these effects constitute serious safety risks, especially if users are driving vehicles or operating machinery (15–17).

Communities, governments and regional organizations have identified substance use as a serious health problem among Inuit population (18–20). Studies on Canada's indigenous peoples reveal that Inuit stand out from southern populations on how they consume alcohol: a lower proportion of them drink daily or every week, and abstinence is common (21–25). However, the data suggest that binge drinking (i.e., 5 drinks or more per occasion) is a common mode of consumption (18). In contrast, little information is available on the use of illicit drugs by indigenous people (15). Despite these data, not much is known about Inuit population. Accordingly, as noted by Scott (26), current studies have not assessed the variability of behaviours according to the diversity of indigenous communities. Moreover, no trend analysis has been performed. This is unfortunate as increased substance use has been noted in other Canadian populations, particularly among youth (27), leading to adjustments in public health priorities. Adequate, up-to-date data on alcohol and drug use are vital to the development of effective health policies and programs for Inuit (15, 20, 28).

For several years, the 1992 Santé Québec Health Survey (SQHS) (29, 30) has been a major source of data on alcohol and drug use in Inuit population living in Nunavik, Northern Quebec (Canada). In 2003, the Nunavik Regional Board of Health and Social Services (NRBHSS) decided to organize an extensive health survey in Nunavik, to track health status and risk factors, resulting in *Qanuippitaa* 2004 (31). Comparing representative data appears to be critical in following the evolution of substance use among Inuit from Nunavik and in determining the necessity of re-evaluating this important health issue. The present article reports alcohol and drug use among Inuit living in Nunavik between the 1990s and 2000s, and identifies socio-demographic characteristics associated with their most recent substance use.

## Methods

### SQHS 1992

The SQHS of the Nunavik Inuit (1992) (29, 30), an extension of the master Quebec Health Survey of 1987, provided a general health and social portrait of this population. It was based on 7 data collection instruments derived from surveys already conducted by Santé Québec among other residents of Quebec. Some questions from the previous surveys were adapted as much as possible to the realities of the north. An editorial committee composed of major partners in the survey supervised questionnaire development: Santé Québec, Inuit, the Quebec Ministry of Health and Social Services (MSSS) and the NRBHSS. The questionnaire was then translated from French to English, and from English to Inuktitut. All translations were reviewed and cross-checked.

The SQHS was conducted in face-to-face interviews and via self-administered questionnaire. All instruments and survey procedures were pretested in 11 Inukjuak households consisting of 30 Inuit of all ages. Nurses went to respondents’ homes and conducted interviews, accompanied by an Inuit interpreter or interviewer able to translate instructions and questions into Inuktitut and respondent answers in English. Average interview length was estimated to be 2 hours.

The population sample covered by the survey comprised 7,078 persons aged 15 years or older spread out in 14 villages (1,567 households), with an average participation rate of 67%. The target population consisted of permanent residents of private Inuit households living within the borders of Nunavik (the territory of Nunavik, the sub-region, the Hudson Bay and Ungava Bay coasts). The MSSS provided major financial support for the survey. Santé Québec coordinated the project, and the Quebec Bureau of Statistics was called upon for its expertise and to furnish advice on sampling and statistical analysis.

### The Nunavik Inuit Health Survey Qanuippitaa (2004)

This survey was conducted among the Nunavik population in 2004 and was approved by the Ethics Committees of Université Laval and Santé Publique du Québec (31, 32). According to the 2001 Canadian census, the 14 communities of Nunavik amounted to 9,632 inhabitants, 91% of whom identified themselves as Inuit. Data were collected on the ice-breaker *Amundsen*, which visited the 14 villages of Nunavik. The study was based on self-administered and interviewer-completed questionnaires. Private Inuit households were visited by an interviewer who met the household respondent to complete an identification chart and a household questionnaire. All individuals of the same household, aged 15 years or older, were invited to meet the survey staff a few days later, on the *Amundsen*, where they completed a confidential, self-administered questionnaire. Participants were required to fill the questionnaire on their own, but many turned to the interviewer/translator to complete part of or the entire questionnaire.

The target population of the survey included permanent residents of Nunavik, except residents of collective dwellings and households in which there were no Inuit aged 18 years or older. The survey undertook stratified random sampling of private Inuit households. Community was the only stratification variable analysed. Stratification provided up-to-standard representation of the target population. Among the 677 households visited by the interviewers, 521 agreed to participate in the survey. A total of 1,056 individuals participated in at least 1 activity of this survey. The confidential questionnaire was completed by 969 of these subjects, 856 adults and 113 minors aged 15 years or over.

### Variables assessed

Questions on lifetime alcohol use and frequency of consumption helped to identify drinker types in both surveys: regular drinkers (individuals consuming alcohol at least once a month), occasional drinkers (individuals consuming alcohol during 12 months preceding the survey but less than once a month), former drinkers (individuals previously consuming alcohol but who had not consumed any during the 12 months preceding the survey), and abstainers (individuals never consuming alcohol) (10, 29, 33). Questions on the quantity of alcohol consumed per occasion and the frequency of heavy drinking episodes (5 drinks or more per drinking day) collected information on the proportion of drinkers who adopted this mode of consumption. The CAGE questionnaire, screening for alcoholism (34), was integrated into the confidential questionnaire. Depending on the population, its sensitivity varied from 43 to 94%, and its specificity, from 70 to 97% (35). A CAGE score of 2 or higher may be a sign of alcohol consumption that risks problematic use. However, it may be pertinent to use a different cut-off point (≥2) in certain populations to safeguard the instrument's specificity. A more severe criterion is desirable for groups that are less sensitive to social desirability (31). This is why the proportions of respondents with scores ≥3 were also examined. Questions of drug use documented proportions of cannabis (marijuana and hashish), cocaine or crack, solvents, hallucinogens and injectable drugs over the previous year. Respondents had to declare if they used any of the preceding substances in the last 12 months (yes/no).

This study provided the most recent portrait of socio-demographic characteristics associated with substance use among Inuit: gender, age (15–19, 20–24, 25–44, 45+ years), marital status (single/married or common law/separated, divorced or widowed), education level (completed elementary school or less/incomplete secondary school/completed secondary school or higher), income (less than $20,000/$20,000–$39,999/$40,000 and over), occupation (work vs. other), and village of residence. The latter variable was divided into 2 regions (Hudson and Ungava) because place of residence could influence life habits. Finally, villages were grouped as alcohol-sale permitting (Kuujjuaq and Kuujjuarapik) or dry communities (all others).

### Analyses

Results from SQHS 1992 and *Qanuippitaa* 2004 on substance use variables were compared separately (alcohol and other substances, most specifically cannabis). Comparative proportion chi-square tests corrected for the design effect, and differences were considered significant when p values were ≤0.05. To benchmark the most recent prevalence rates of substance use by Inuit with the overall Canadian population, the results of *Qanuippitaa* 2004 were also compared to the 2003 Canadian Community Health Survey (CCHS) (36) for all of Quebec and Canada when the questions were considered equivalent. Given the sampling procedures in different surveys, these comparisons included adjustment of proportions or rates and taking changes in the population's age structure into account. This adjustment was made on the basis of 5-year age groups, employing the Nunavik 2001 census of Statistics Canada as the reference population for comparison with 1992 survey and 1996 Canada census data for Quebec and Canada. However, only raw data are reported in the text and tables to avoid any possible confusion with adjusted proportions. Moreover, comparisons with these surveys included adjustment for survey design (37). Finally, a few comparisons were made with published data from the Canadian Addiction Survey (CAS) (2) to ascertain substance use by Inuit population vs. the general Canadian population. However, no adjustments were made for these latter comparisons because of lack of access to the CAS 2004 database.

We finally compared proportions of lifetime alcohol consumption and cannabis use in the preceding year according to socio-demographic characteristics indexed in *Qanuippitaa* 2004 with chi-square tests corrected for the design effect and significant p values ≤0.05. When we analysed other substance use rates according to socio-demographic characteristics, the coefficient of variation (CV) quantified the accuracy of estimates, with qualification by the Statistics Canada Scale. A superscript “E” next to an estimate indicates a marginal estimate (CV between 16.6 and 33.3%). Estimates with unreliable levels of accuracy (CV>33.3%) are not presented and replaced by superscript “F.”

## Results

### Alcohol consumption

**Fig. 1:**
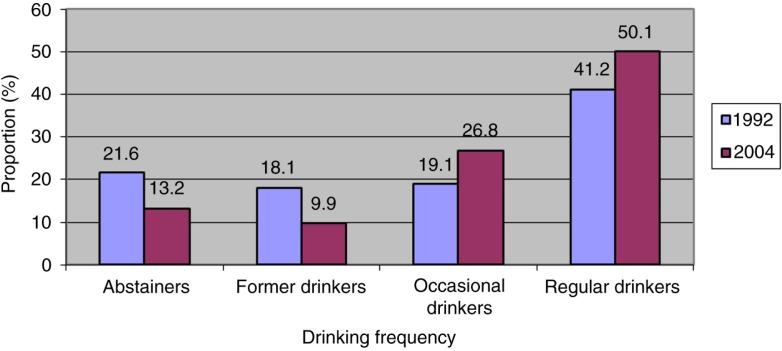
Comparison of drinking frequency in preceding year (%) in Inuit population aged 15 years or over in Nunavik, 1992 and 2004. Sources: Santé Québec Health Survey 1992 and Nunavik Inuit Health Survey 2004.

In 1992 and 2004, alcohol was the substance used by the largest proportion of Inuit study participants: 60.3% in 1992 and 76.9% in 2004 reported having drunk alcohol in the year preceding the survey; 41.2% were regular drinkers in 1992, and 50.1% in 2004 (Fig. 1). Between SQHS 1992 and *Qanuippitaa* 2004, drinking prevalence increased by 16.6% in the year preceding the survey in Nunavik. 63.6% of Nunavik drinkers reported at least 1 episode of heavy drinking in the previous year in 1992 compared to 88.8% in 2004.

Taking a CAGE score of 2 or more as cut-off, 51.6% of participants who had consumed alcohol in the year preceding SQHS 1992 and 50.8% in the year preceding *Qanuippitaa* 2004 were considered to be at risk of repercussions in their lives as a result of alcohol (data not shown). Adopting a more severe CAGE criterion in *Qanuippitaa* 2004, that is, a score of 3 or more, drinking level was likely to have repercussions in the daily lives of 25.7% of drinkers.

Comparisons between Qanuippitaa 2004 and CCHS 2003 revealed that, in Nunavik, the proportion of drinkers (76.9%) was significantly lower than that observed in Canada as a whole (80.5%; p<0.0001) and Quebec (84.9%; p<0.0001). It was also lower than the proportion in Quebec in CAS 2004 (82.3%).

**Fig. 2:**
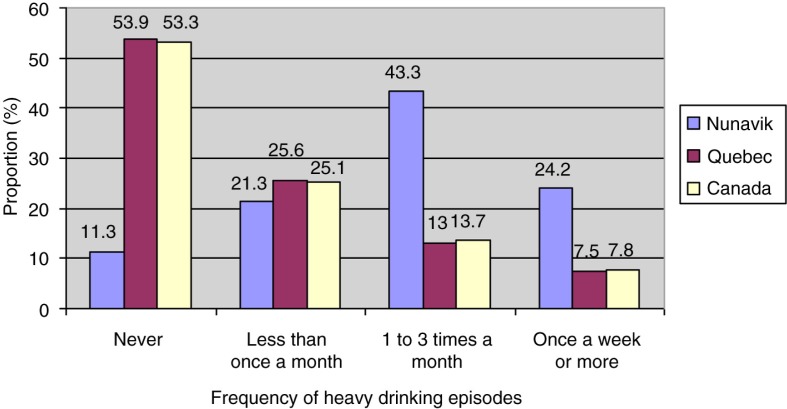
Frequency of heavy drinking episodes in preceding year (%) in Inuit population aged 15 years or over: comparison of the populations of Nunavik 2004, southern Quebec 2003 and Canada 2003. Sources: Nunavik Inuit Health Survey 2004 and Canadian Community Health Survey 2003.

When we compared results from *Qanuippitaa* 2004 with other Canadian populations, the proportion of drinkers reporting having had at least 1 episode of heavy drinking in the previous year in Nunavik (88.8%) was twice as high as the rates in CCHS 2003 for southern Quebec (46.1%) and Canada as a whole (46.7%) (Fig. 2). Weekly heavy drinking in Nunavik (24.2%) was 3 times higher than the rates among Quebecers (7.5%) and Canadians (7.8%).

When we looked at tendencies of substance use behaviours in both Inuit surveys (1992 and 2004) according to socio-demographic variables, the proportion of alcohol users was significantly higher among men than women and among participants under 45 years of age (for more information about results of the SQHS, see Jetté and Thibeault (30). In *Qanuippitaa* 2004 (Table I), we also observed a higher proportion of drinkers among highly educated participants, with an annual income over $20,000 and who had a job. At that time, the proportion of drinkers was about 10% higher in villages where alcohol sales were permitted (Kuujjuaq and Kuujjuaraapik) compared to the rest of Nunavik. Frequencies of heavy drinking episodes were: never 11.3%; less than once a month 21.3%; 1–3 times a month 43.3%; at least once a week 24.2%. There were no significant differences in frequencies of heavy drinking episodes by gender. Finally, the proportion of individuals at risk of problematic alcohol use evaluated by CAGE measurement was significantly higher among women (55.1% vs. 47.1% among men, p=0.03) and in the 25–44-year-old age group (57.1%, p=0.008), compared to the 15–24 and 45 and older age groups (45.6 and 43.9%, respectively).

**Table I:** Prevalence of lifetime alcohol consumption (%) in Inuit population aged 15 years or over in Nunavik, 2004

	EP^a^	Partial non-response (%)	Consumers		p^b^

			%	95% CI	
Gender					0.005
Men	2,710	3.4	89.6	86.5–92.1	
Women	2,420	5.9	84.0	81.3–86.7	
Age group					<0.0001
15–24 years	1,670	4.0	87.9	83.9–91.2	
25–44 years	2,380	2.9	91.2	88.1–93.7	
45 years +	1,070	8.5	77.0	71.7–82.3	
Marital status					0.059
Single	2,010	4.7	87.2	83.4–90.5	
Married or common law	2,750	4.4	88.2	85.3–90.6	
Separated, divorced or widowed	300	6.5	77.4	64.2–87.6	
Education level^c^					<0.0001
Completed elementary school or less	900	10.6	71.9	65.1–78.0	
Incomplete secondary school	2,930	3.2	89.7	87.5–92.0	
Completed secondary school or higher	1,150	1.0	93.4	89.2–96.3	
Income					0.002
Less than $20,000	2,500	4.6	85.4	82.2–88.2	
$20,000–39,999	1,100	4.3	92.5	88.0–95.6	
$40,000 and over	870	1.7	92.7	88.2–95.9	
Occupation					<0.0001
Work	3,580	3.1	90.8	88.5–92.8	
Other^d^	1,340	6.0	79.8	75.3–84.2	
Region					0.002
Hudson	2,840	6.7	84.2	80.7–87.3	
Ungava	2,290	1.8	90.4	87.8–92.5	
Type of community					<0.0001
Dry communities	3,800	5.9	84.5	82.1–87.0	
Alcohol sales permitted	1,320	0.5	94.0	90.2–96.7	

a Estimated number of Nunavik residents in this situation, according to prevalence rates and the sampling methods used.

b Chi-square test with p values.

c The number of people with post-secondary education is likely to be overestimated.

d Other: hunter support program, housework, retired or on pension, unemployment insurance, social welfare, student or other (disability, maternity leave, etc.).

Source: Nunavik Inuit Health Survey 2004.

### Drug use

**Fig. 3:**
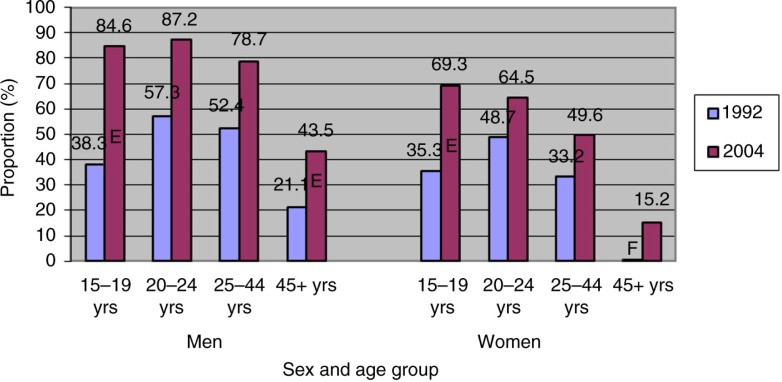
Proportions of illicit drug users in preceding year by gender and age group (%) in Inuit population aged 15 years or over in Nunavik, 1992 and 2004. ^E^Interpret with circumspection ^F^Unreliable estimate. Publication forbidden Sources: Nunavik Inuit Health Survey 2004 and Santé Québec Health Survey 1992.

Six (60.3%) of 10 respondents stated they had used at least 1 illicit drug in the 12 months preceding *Qanuippitaa* 2004. This proportion is clearly higher than in SQHS 1992 (36.5%, p<0.0001). It was also 4 times higher than in CAS 2004 for the rest of Canada (14.5%) (2). Comparison of the results with SQHS 1992 revealed that the proportion of drug users increased as much among women as among men in all age groups (Fig. 3).

In both Inuit surveys, cannabis (marijuana and hashish combined) was the most frequently used drug among respondents, with rates of 55.5% in 1992 and 60.2% in 2004. Table II describes the socio-demographic characteristics of cannabis users among the most recent data from *Qanuippitaa* 2004. As in 1992 (66.4% of men and 44.9% of women), men in 2004 were significantly more prevalent cannabis users than women. Cannabis use was also significantly more frequent among young people. Although nearly 8 out of 10 participants aged 15–19 years were cannabis users, this practice was also common among older men and women: (a) men: 15–19 years old, 84.6%; 20–24 years old, 87.2%; 25–44 years old, 79.0%; (b) women: 15–19 years old, 69.9%; 20–24 years old, 63.3%; 25–44 years old, 49.9%. The prevalence of cannabis use in Nunavik increased since 1992, with current users estimated to be 38.3% of the population. It was particularly significant among the youth (15–19 years), with cannabis use going from 38 to 78% of the population in 12 years. There were about 4 times more cannabis users in Nunavik than in southern Quebec (15.8%) and Canada as a whole (14.1%) (1).

**Table II:** Prevalence of cannabis use in preceding year (%) in Inuit population aged 15 years or over in Nunavik, 2004

	EP^a^	Partial non-response (%)	Consumers		p^b^

			%	95% CI	
Gender					<0.0001
Men	2,200	0.4	72.6	68.8–76.5	
Women	1,350	2.0	46.8	43.1–50.5	
Age group					<0.0001
15–19 years	830	1.6	77.7	71.2–83.3	
20–24 years	630	0.7	75.5	67.9–82.1	
25–44 years	1,690	1.2	65.0	60.4–69.5	
45 years +	400	1.1	28.6	22.9–34.3	
Marital status					<0.0001
Single	1,690	1.3	73.1	69.0–77.1	
Married or common law	1,630	1.0	52.5	47.9–57.0	
Separated, divorced or widowed	170	2.6	43.5	30.4–56.5	
Education level^c^					<0.0001
Completed elementary school or less	430	1.8	34.7	27.8–41.5	
Incomplete secondary school	2,350	1.1	72.2	68.5–75.9	
Completed secondary school or higher	690	0.5	56.2	48.9–63.5	
Income					0.0004
Less than $20,000	1,960	1.1	66.9	63.0–70.9	
$20,000–39,999	660	1.0	55.6	48.7–62.5	
$40,000 and over	480	0.6	51.6	43.6–59.6	
Occupation					0.006
Work	2,510	1.1	63.7	60.1–67.3	
Other^d^	910	1.4	54.6	49.1–60.0	
Region					0.583
Hudson	2,000	1.4	59.4	55.6–63.3	
Ungava	1,550	1.0	61.1	56.4–65.9	
Type of community					0.893
Dry community	2,700	0.8	60.0	56.6–63.4	
Alcohol sales permitted	850	2.6	60.6	53.3–67.9	

a Estimated number of Nunavik residents in this situation, according to prevalence rates and the sampling methods used.

b Chi-square test with p values.

c The number of people with post-secondary education is likely to be overestimated.

d Other: hunter support program, housework, retired or on pension, unemployment insurance, social welfare, student or other (disability, maternity leave, etc.).

Source: Nunavik Inuit Health Survey 2004.

After cannabis, cocaine was the most frequently used drug in 2004: 7.5% of respondents stated that they were users in the year preceding *Qanuippitaa* 2004. Other drugs used in the same period were solvents, hallucinogens and injectables, with rates of 5.9, 2.7 and 2.0%, respectively. Cocaine use rates were comparable between men and women but significantly higher (p=0.002) among younger participants: 12.1%^E^ in the 15–19-year-old group and 10.6%^E^ in the 20–24-year-old group vs. 7.3%^E^ in the 24–44 age group. Inhaling solvents was also more frequent among young people (p<0.0001): 13.5%^E^ in the 15–19-year-old group and 11.0%^E^ in the 19–24-year-old group vs. 3.9%^E^ in the 25–44-year-old group. Across Nunavik, the proportions of cocaine and solvent users increased by 50 and 100% respectively, over the last decade (cocaine: 5.1% in 1992 vs. 7.5% in 2004, p=0.05; solvents: 3.0% in 1992 vs. 5.9% in 2004, p=0.001).

The proportion of Nunavik residents who used cocaine in *Qanuippitaa* 2004 was 3 times higher than in CAS 2004 for Canada as a whole (2). Notably, the proportion of solvent users was 7 times higher in communities where alcohol sales were not permitted, compared to communities where alcohol was sold. Because SQHS 1992 did not document the use of hallucinogens or injectable drugs, it was impossible to determine trends for these drugs over time. The low rates reported for hallucinogens and injectables do not permit comparisons based on socio-demographic characteristics. Finally, drug and alcohol consumption were closely related to each other among Inuit. About 60% of drug users during the 12 months preceding *Qanuippitaa* 2004 consumed alcohol on a regular basis, whereas 35.5% of non-drug users did so. Only 7.2% of drug users were alcohol abstainers, while 22.8% of non-drug users were alcohol abstainers.

## Discussion

The main objective of this article was to follow the evolution of alcohol and drug use in the Nunavik Inuit population. It indicates that alcohol and drug use among Nunavik Inuit increased between 1992 and 2004 and may diverge from our knowledge of other drinking and substance use cultures. In comparison to southern populations for 2004, Inuit were characterized by a lower proportion of lifetime drinkers. In contrast, long-term trends in prevalence of alcohol use in the general Canadian population have remained stable over time: approximately 80–85% of men and 70–75% of women since 1989 (38). In Inuit population, the prevalence of alcohol use increased substantially over time. Moreover, significantly more alcohol was consumed per drinking episode. This was in accordance with Korhonen (18), who claimed that Inuit population tends to drink occasionally, but more intensely. Heavy drinking was not only common among Inuit drinkers, but also occurred on a weekly basis in almost a quarter of the population. In contrast, the proportion of the population considered to be at risk of alcohol-induced repercussions in their lives appears to have remained stable between 1992 and 2004.

Cannabis was by far the most prevalent illicit drug in Nunavik, with more users in 2004 than in 1992. Its use was generalized among younger adult males, but was also very frequent in older Inuit irrespective of gender. Unlike alcohol, increased cannabis use has been noted between the beginning of the 1990s and 2000s, particularly among youth in Canada (38). Since 2008, however, we have seen a general decrease of cannabis use among the general Canadian population and youth (39). Overall, such increases among Inuit may reflect lower perceived harm related to cannabis, as seen in other parts of North America (40). Hall and Degenhardt (41) and Lynskey and Hall (42) noted that cannabis may be used for its mood-altering and relaxation properties, or encouraged by peers in the social context. Although less frequent, consumption of other illicit drugs, such as cocaine, has increased significantly between the 1992 and 2004 surveys. This trend has also been observed among the general Canadian population, but young Canadians report that cocaine use has been declining since 2010 (39). The situation is disconcerting, particularly if the proportion of drug users keeps growing at the same rate over time (43, 44).

Finally, our study indicates that the youth population of Nunavik is especially prone to the concomitant use of alcohol and illicit drugs with no difference between genders. Substance use by women of childbearing age is of particular concern as they may be unaware of the dangers or believe that these teratogens have a low impact on their newborns (45, 46).

## Conclusion

The results of this analysis emphasize the need for culturally-adapted public health prevention and intervention programs to decrease problematic substance use among Inuit. Our results reinforce the need to document substance use over time to establish whether prevalence continues to increase or not with the rapid societal changes occurring in this population.
